# Association Between Second-Hand Smoke Exposure and Respiratory Symptoms Among the General Population of Non-smoker Adults in Saudi Arabia: A Cross-Sectional Study

**DOI:** 10.7759/cureus.49243

**Published:** 2023-11-22

**Authors:** Mohammed Madkhali, Safa Shariff, Raghad Albalawi, Abdulrahman Aqeel, Abdulqader Alshahrani, Raghd Alabdullah, Raghad Alasmari, Wajd Aljohani

**Affiliations:** 1 Faculty of Medicine, Jazan University, Jazan, SAU; 2 Faculty of Medicine, Al-Rayan Colleges, Madinah, SAU; 3 Internal Medicine, Armed Forces Hospital South Region, Khamis Mushait, SAU; 4 Faculty of Medicine, Umm Al-Qura University, Makkah, SAU; 5 Faculty of Medicine, Taif University, Taif, SAU; 6 Internal Medicine, King Abdullah Medical Complex - Jeddah, Jeddah, SAU

**Keywords:** public health and safety, saudi arabia, respiratory symptoms, passive smoking, second-hand smoke

## Abstract

Background: Second-hand smoke (SHS) exposure is associated with respiratory symptoms such as cough, dyspnea, and wheezing. However, data on this association in Saudi Arabia is limited.

Objectives: The objective of this study is to investigate the prevalence of SHS exposure and its association with respiratory symptoms among non-smoker adults in Saudi Arabia.

Methods: Data collection was carried out over the course of two months by distributing an online questionnaire among adults living in Saudi Arabia. The survey consisted of questions assessing sociodemographic factors, SHS exposure, and the presence of respiratory symptoms. Statistical analysis was performed using IBM SPSS Statistics for Windows, Version 22 (Released 2013; IBM Corp., Armonk, New York, United States). Data was considered significant if p<0.05.

Results: The questionnaire was completed by 1360 participants. Most were females (n=845, 72.1%) and individuals aged 18 to 30 years (n=838, 71.5%). From the analyzed records, 67.3% (n=789) reported SHS exposure. Among them, 40.3% (n=472) reported SHS exposure from household sources, 32.6% (n=382) from work colleagues, and 30.5% (n=357) from friends. The majority (n=306, 76.9%) of those exposed at home reported daily SHS exposure. The main source of home exposure was male family members, particularly the father (n=201, 42.6%). The majority (n=985, 84.0%) of participants reported not having any kind of mold or damage at their place of residence. Individuals with SHS exposure were more likely to report asthma (p=0.043), chest whistling or wheezing (p=0.021), chronic cough (p<0.001), productive cough (p<0.001), and nasal symptoms without a cold (p<0.001). These individuals also demonstrated a higher average symptom score than those not exposed to SHS.

Conclusion: The study reveals that a significant percentage of the Saudi population is exposed to SHS daily, mainly from household sources, especially male family members. A significant association was found between SHS exposure and the presence of respiratory symptoms. Public awareness regarding the prevalence and dangers of SHS exposure is essential in order to alleviate the impact of SHS on the health of the general Saudi population. Additionally, further research is required in this field and demographic group to develop appropriate interventions.

## Introduction

Active smoking has well-established harmful effects [1]. However, second-hand smoke (SHS), which contains a mixture of respiratory irritants and carcinogens, also contributes to significant health issues in non-smokers [2,3]. According to the CDC, SHS exposure can cause coronary heart disease, stroke, and even premature death [4]. The same CDC article mentions that SHS can cause low birth weight in babies born from pregnant women exposed to SHS, respiratory infections, asthma attacks, and even sudden infant death syndrome (SIDS) in children. It also establishes that since 1964, around 2,500,000 non-smokers died from illnesses caused by SHS exposure. Yet, the most common signs of SHS-related morbidity are respiratory symptoms such as coughing, wheezing, and dyspnea [5].

Although SHS has an established negative impact on health worldwide, regional variations in these effects may occur as a result of socioeconomic, cultural, and demographic factors [6]. Saudi Arabia is presently undergoing a significant social and economic change. As a result, smoking and SHS exposure patterns are also changing. Tobacco use is ingrained in the Arabian culture, particularly the use of water pipes (shisha) [7]. Recently however, there have been more awareness campaigns and programs aiming to reduce the prevalence of smoking, especially among women and young people [8]. In spite of these initiatives, smoking is still a major public health issue in Saudi Arabia [9]. These facts lead us to hypothesize that adult non-smokers may be at a higher risk of SHS exposure, which may increase the risk of respiratory symptoms in this group. However, actual data describing this association within the specific demographic of Saudi Arabia is limited.

By investigating the association between SHS exposure and respiratory symptoms in Saudi Arabia's general population of non-smoking adults, this study seeks to close this gap in the literature and offer insights regarding the health effects of SHS exposure in this particular demographic. Developing targeted interventions and policies to protect the health and well-being of the population requires understanding the risks that non-smoker adults face from SHS. The findings gained from this study may add to the global body of knowledge on SHS-related health impacts by providing comparative data for cross-national assessments.

## Materials and methods

This cross-sectional study was conducted from June 2023 to July 2023, and the study population included all citizens and residents of Saudi Arabia above 18 who have been exposed to second-hand smoke. The inclusion criteria were all adults exposed to SHS who were living in Saudi Arabia, mentally fit, and willing to participate. The exclusion criteria were population outside Saudi Arabia, mentally unwell, smokers, and children. Participants who did not consent to the survey and those who did not complete the questionnaire were also excluded from the study. The sample size of this study was calculated as 385 by using the following formula for probability sampling:

$N= Z21-&alpha;/2 P(1-P)/d2$

Where N is the sample size, Z is the standard normal distribution (1.96 to a confidence level of 95%), P is the anticipated population proportion (50%) for the maximum sample size, and D is an error not more than 0.05 (5%). Considering 10% non-response rate, the required sample size was 425 participants. A total of 1360 responses were obtained which was about three times the required sample size. A convenience sampling technique was used.

A self-administered semi-structured questionnaire extracted from a similar study conducted in Norway was used for collecting the data [10]. The questionnaire consisted of three parts. At the very beginning, consent was gained by the first survey item, "Do you agree to participate in this study" followed by yes or no options. Regarding the content of the survey itself, the first part was socio-demographic information of the participants such as age, gender, nationality (Saudi, non-Saudi), residency, marital status, educational level, and occupation. The second part consisted of questions measuring the extent of SHS exposure in the home or at work. Smoking exposure at home was assessed by the survey item, “Does anyone smoke tobacco in your current home?” followed by frequency options of almost daily; 1-4 times/week; 1-3 times/month; or never. Childhood SHS exposure was defined by an affirmative answer to any one of three questions: “Was your mother a regular smoker when you were a child?”; “Was your father a regular smoker when you were a child?”; or “Were there any other regular smokers in your home when you were a child?”. SHS exposure at work was assessed by the question: “Have you been exposed to gas, smoke, dust, or fumes in your work?”. Exposure to mold and dampness at home was assessed by an affirmative response to one of the following: “Have you had any of the following in your residence: water damage/damage from dampness inside the dwelling on walls, floors or ceilings, warped plastic mats, yellowed plastic coating or wood flooring that has become dark due to moisture; or visible mold on walls, floors or ceilings?”. Only those who affirmed this exposure during the past 12 months were categorized as exposed. The third part contained questions assessing the presence of respiratory symptoms to define respiratory health outcomes. Respiratory symptoms assessed included: asthma, wheezing, chronic cough, productive cough, nasal symptoms, shortness of breath, and use of asthma medication/inhalers. The study protocol was approved, and official permissions were obtained from the Research Ethics Committee of the College of Medicine of Jazan University with approval number REC-44/10/641. The questionnaire was distributed online via data collectors across social media platforms in Saudi Arabia.

SHS exposure is a composite variable defined as the exposure of participants to smoke at home, at the workplace, or due to contact with a friend. Each reported symptom was assigned 1, and an overall symptoms score was calculated by summing up the values of symptoms reported by the participants over the past 12 months (seven symptoms) and the use of medications for respiratory symptoms. These eight variables gave rise to a total score ranging between 0 (no symptoms) and 8 (all symptoms).

Data was coded, entered, and analyzed using IBM SPSS Statistics for Windows, Version 22 (Released 2013; IBM Corp., Armonk, New York, United States). The association between respiratory symptoms and SHS exposure was assessed using an inferential analysis using a Pearson's Chi-squared test, whereas the difference in the symptoms score between those with positive or negative SHS exposure was assessed using a Wilcoxon rank sum test. Significantly associated variables from the inferential analysis were subsequently incorporated in multivariable logistic regression models to assess the role of SHS exposure as a risk factor for respiratory symptoms (each symptom was entered as a dependent variable in a separate model). Independent variables included SHS exposure, sociodemographic characteristics, and exposure to mold and dampness at home. Results of the logistic regression analysis were expressed as odds ratios (ORs) and 95% confidence intervals (95% CIs). We assessed the role of SHS exposure on the symptoms score in a multivariable generalized linear model using the same covariates. The results of SHS exposure were exclusively expressed in the respective tables. Statistical significance was deemed at p < 0.05.

## Results

Sociodemographic characteristics of the participants

We received a total of 1360 responses on the online platform. However, we excluded 18 records of those who disagreed to participate and 170 records of smokers. Therefore, we analyzed the records of 1172 participants. The majority of participants were females (n=845, 72.1%) and aged between 18 and 30 years (n=838, 71.5%). Saudi nationals constituted most of the participants (n=1,026, 89.2%), primarily residing in the southern region (n=326, 27.8%). Most participants were single (n=752, 64.2%) and held a bachelor's degree (n=686, 58.5%). In terms of employment, a significant portion were students (n=590, 50.3%), followed by employed individuals (n=326, 27.8%). The sociodemographic characteristics of the participants are shown in Table 1.

**Table 1:** Sociodemographic characteristics of the participants (n=1172)

Characteristic	N (%)
Gender	
Male	327 (27.9%)
Female	845 (72.1%)
Age (Years)	
18 to 30	838 (71.5%)
31 to 40	149 (12.7%)
41 to 50	126 (10.8%)
>50	59 (5.0%)
Nationality	
Saudi	1,046 (89.2%)
Non-Saudi	126 (10.8%)
Province	
Eastern region	181 (15.4%)
Western region	287 (24.5%)
Northern region	168 (14.3%)
Southern region	326 (27.8%)
Central region	210 (17.9%)
Marital Status	
Single	752 (64.2%)
Married	399 (34.0%)
Other	21 (1.8%)
Educational level	
Primary school	20 (1.7%)
Middle school	24 (2.0%)
High school	204 (17.4%)
University student	174 (14.8%)
Bachelors	686 (58.5%)
Higher studies	64 (5.5%)
Employment status	
Student	590 (50.3%)
Employed	326 (27.8%)
Unemployed	215 (18.3%)
Retired	41 (3.5%)

Characteristics of SHS exposure

**Figure 1:**
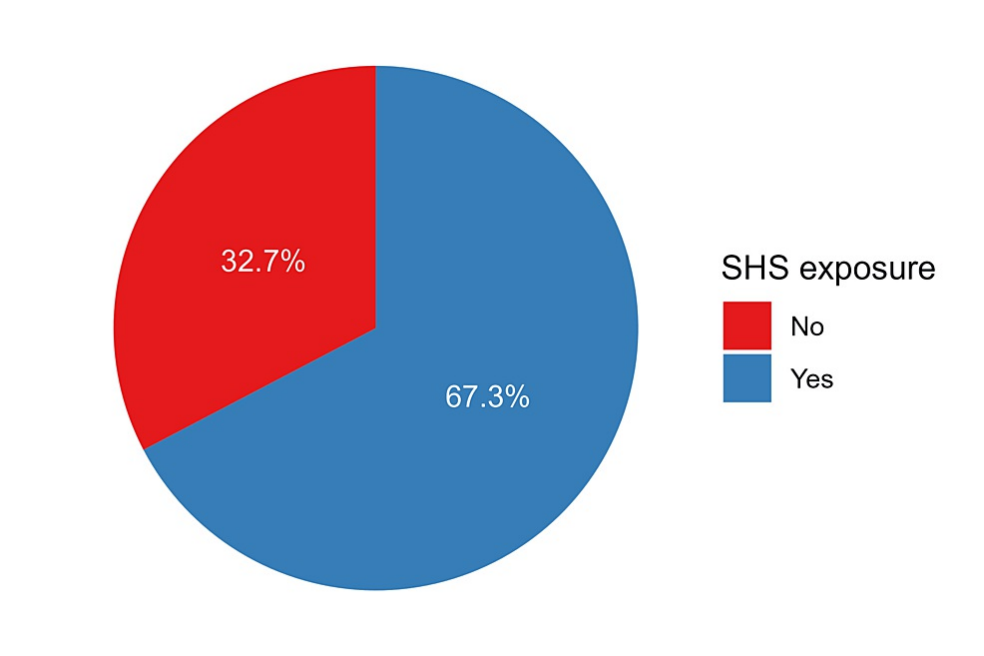
The proportions of participants based on the SHS exposure status SHS: Second-hand smoke

**Figure 2:**
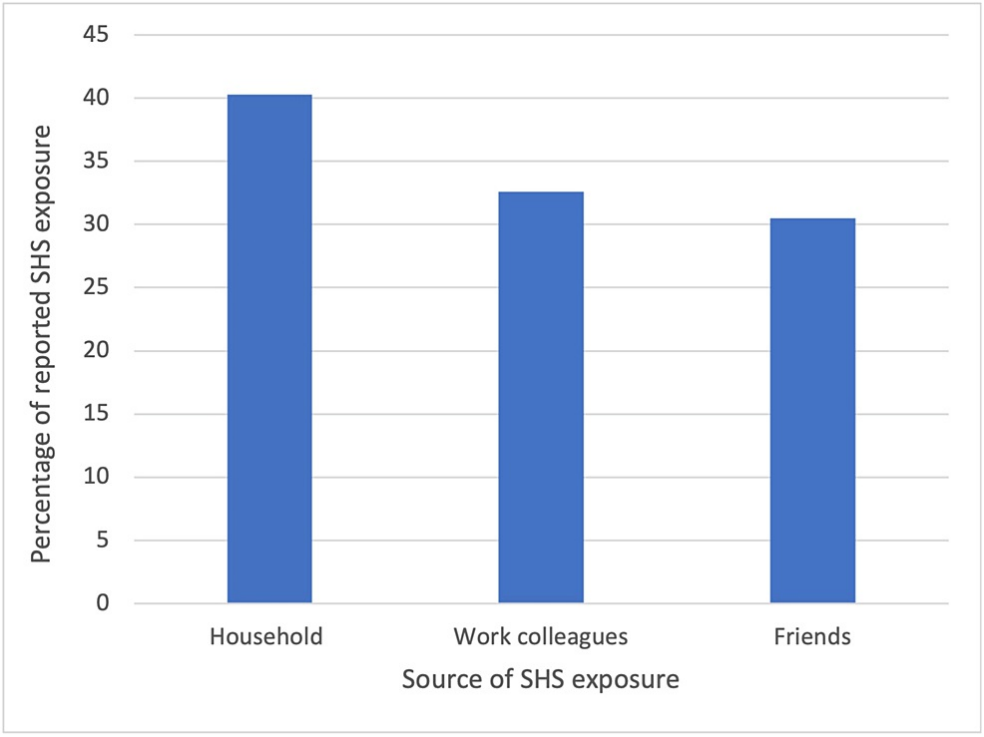
The proportions of sources of SHS exposure SHS: Second-hand smoke

Out of the participants under study, 789 (67.3%) respondents reported exposure to SHS (Figure 1). SHS exposure was more frequent at home (n=472, 40.3%), from work colleagues (n=382, 32.6%), or friends (n=357, 30.5%). These results are illustrated in Figure 2. Regarding second-hand smoke (SHS) exposure at home, among the 472 participants, the majority reported experiencing SHS exposure almost daily (n=306, 76.9%). Moreover, the prevalence of maternal regular smoking during participants' childhood was low (n=13, 2.8%), while a relatively higher percentage indicated that their father was a regular smoker during their childhood (n=201, 42.6%). Additionally, a substantial proportion of participants noted the presence of other regular smokers at home during their childhood (n=230, 48.7%). The prevalence of various individuals in this category included brothers (n=51, 26.7%), uncles (n=15, 7.9%), and to a lesser extent, husbands (n=2, 1.0%) and grandparents (n=4, 2.1%). The characteristics of SHS exposure at home are shown in Table 2.

**Table 2:** Characteristics of SHS exposure at home (n=472) SHS: Second-hand smoke; N: number of samples *The variable had 74 missing records §Descriptive statistics are based on 230 responses from participants who reported other regular smokers at home (with 39 missing records).

Characteristic	N (%)
Frequency of SHS exposure at home*	
1 to 3 times per month	30 (7.5%)
1 to 4 times per week	62 (15.6%)
Almost daily	306 (76.9%)
Mother was a regular smoker when you were a child	
No	459 (97.2%)
Yes	13 (2.8%)
Father was a regular smoker when you were a child	
No	271 (57.4%)
Yes	201 (42.6%)
Other regular smokers in your home when you were a child	
No	242 (51.3%)
Yes	230 (48.7%)
Brother§	51 (26.7%)
Uncle§	15 (7.9%)
Husband§	2 (1.0%)
Grandfather/Grandmother§	4 (2.1%)

Characteristics of exposure to mold and dampness at home

Concerning exposure to mold and dampness within the home environment, among the participants (n=1172), the majority reported not having any kind of damage in their place of residence (n=985, 84.0%). However, 187 (16.0%) participants indicated the presence of various types of damage, including dampness-related issues. Specifically, 58 (61.1%) participants reported damage from dampness inside the dwelling on walls, floors, or ceilings, and a smaller percentage noted visible mold on these surfaces (n=18, 18.9%). Furthermore, 226 (19.3%) participants reported signs of moisture damage, water leakage, or mildew in their homes over the past 10 years. Among this group, 33 (32.7%) reported moisture damage, 23 (22.8%) reported water leakage, and 19 (18.8%) reported mildew (Table 3).

**Table 3:** Characteristics of exposure to mold and dampness at home ¥Damage types included water damage/damage from dampness inside the dwelling on walls, floors or ceilings, warped plastic mats, yellowed plastic coating or wood flooring that has become dark due to moisture; or visible mold on walls, floors or ceilings *Descriptive statistics are based on 187 responses (92 missing records) §Descriptive statistics are based on 226 responses (125 missing records)

Characteristic	N (%)
Having kinds of damage in the place of residence¥	
No	985 (84.0%)
Yes	187 (16.0%)
Damage from dampness inside the dwelling on walls, floors or ceilings*	58 (61.1%)
Visible mold on walls, floors or ceilings*	18 (18.9%)
Yellowed plastic coating*	6 (6.3%)
Having signs of moisture damage, water leakage, or mildew in your home over the past 10 years	
No	946 (80.7%)
Yes	226 (19.3%)
Water leakage§	23 (22.8%)
Moisture damage§	33 (32.7%)
Mildew§	19 (18.8%)

The association between SHS exposure and respiratory symptoms

Individuals with SHS exposure were more likely to report the following symptoms: asthma (p=0.043), chest whistling or wheezing (p=0.021), chronic cough (p<0.001), productive cough (p<0.001), and nasal symptoms without a cold (p<0.001), as well as the following symptoms experienced during the past 12 months: cough (p<0.001), dyspnea (p=0.001), nocturnal chest tightness (p=0.006), wheezing or whistling in the chest (p=0.017). Furthermore, SHS exposure was significantly associated with the use of asthma medications (p=0.002). Additionally, participants exposed to SHS had a higher average symptoms score (1.2±1.5) compared to those with no SHS exposure (0.8±1.3, p<0.001, Table 4).

**Table 4:** The association between SHS exposure and respiratory symptoms SHS: Second-hand smoke; N: number of samples

Characteristic	SHS exposure N (%)		p-value
	No N=383(32.7%)	Yes N=789(67.3%)	
Ever had symptoms			
Asthma			0.043
No	346 (33.7%)	680 (66.3%)	
Yes	37 (25.3%)	109 (74.7%)	
Physician-diagnosed asthma			0.109
No	348 (33.5%)	692 (66.5%)	
Yes	35 (26.5%)	97 (73.5%)	
Chest whistling or wheezing			0.021
No	299 (34.6%)	566 (65.4%)	
Yes	84 (27.4%)	223 (72.6%)	
Chronic cough			<0.001
No	302 (36.1%)	534 (63.9%)	
Yes	81 (24.1%)	255 (75.9%)	
Productive cough			<0.001
No	343 (35.2%)	632 (64.8%)	
Yes	40 (20.3%)	157 (79.7%)	
Nasal symptoms without cold			<0.001
No	212 (41.1%)	304 (58.9%)	
Yes	171 (26.1%)	485 (73.9%)	
Experiencing symptoms over the past 12 months			
Asthma attack			0.497
No	371 (32.9%)	758 (67.1%)	
Yes	12 (27.9%)	31 (72.1%)	
Cough			<0.001
No	284 (36.3%)	499 (63.7%)	
Yes	99 (25.4%)	290 (74.6%)	
Dyspnea with wheezing			0.175
No	357 (33.2%)	717 (66.8%)	
Yes	26 (26.5%)	72 (73.5%)	
Wheezing without a cold			0.099
No	353 (33.4%)	703 (66.6%)	
Yes	30 (25.9%)	86 (74.1%)	
Dyspnea			0.001
No	323 (35.0%)	600 (65.0%)	
Yes	60 (24.1%)	189 (75.9%)	
Nocturnal chest tightness			0.006
No	352 (34.0%)	682 (66.0%)	
Yes	31 (22.5%)	107 (77.5%)	
Wheezing or whistling in the chest			0.017
No	352 (33.8%)	688 (66.2%)	
Yes	31 (23.5%)	101 (76.5%)	
Use of asthma medications			0.002
No	371 (33.8%)	728 (66.2%)	
Yes	12 (16.4%)	61 (83.6%)	
Symptoms score	0.8 ± 1.3	1.2 ± 1.5	<0.001

SHS exposure as a risk factor for respiratory symptoms

Notably, individuals exposed to SHS exhibited higher odds of reporting the following symptoms: asthma (OR=1.65, 95% CI 1.11 to 2.52, p=0.016), chest whistling or wheezing (OR=1.42, 95% CI 1.06 to 1.93, p=0.021), chronic cough (OR = 1.83, 95% CI 1.36 to 2.48, p < 0.001), productive cough (OR=2.42, 95% CI 1.65 to 3.61, p<0.001), nasal symptoms without a cold (OR=2.02, 95% CI 1.56 to 2.62, p<0.001), use of asthma medications (OR=3.28, 95% CI 1.74 to 6.68, p<0.001), cough over the past 12 months (OR=1.71, 95% CI 1.29 to 2.27, p<0.001), dyspnea (shortness of breath) over the past 12 months (OR=1.76, 95% CI 1.26 to 2.49, p=0.001), nocturnal chest tightness over the past 12 months (OR=2.00, 95% CI 1.31 to 3.14, p=0.002), and wheezing or whistling in the chest over the past 12 months (OR=1.72, 95% CI 1.12 to 2.71, p=0.015). Furthermore, SHS exposure was a significant risk factor for an increased symptoms score (beta=0.43, 95%CI, 0.26 to 0.61, p<0.001, Table 5).

**Table 5:** Results of the multivariable regression models for SHS exposure as a risk factor for respiratory symptoms SHS: Second-hand smoke; N: number of samples; OR: odds ratio; CI: confidence interval *Variable results are expressed as beta coefficients and 95% confidence intervals; otherwise, results are expressed as odds ratios and 95%CIs.

Parameter	OR	95% CI	p-value
Asthma	1.65	1.11, 2.52	0.016
Chest whistling or wheezing	1.42	1.06, 1.93	0.021
Chronic cough	1.83	1.36, 2.48	<0.001
Productive cough	2.42	1.65, 3.61	<0.001
Nasal symptoms without cold	2.02	1.56, 2.62	<0.001
Use of asthma medications	3.28	1.74, 6.68	<0.001
Cough over the past 12 months	1.71	1.29, 2.27	<0.001
Dyspnea over the past 12 months	1.76	1.26, 2.49	0.001
Nocturnal chest tightness over the past 12 months	2.00	1.31, 3.14	0.002
Wheezing or whistling in the chest over the past 12 months	1.72	1.12, 2.71	0.015
Symptoms score*	0.43	0.26, 0.61	<0.001

Exposure to mold and dampness as a risk factor for respiratory symptoms

Based on the multiple regression analysis, the exposure to mold and dampness was a significant risk factor for chest whistling or wheezing (OR=1.74, 95% CI 1.13 to 2.67, p = 0.011). No other respiratory symptoms were predicted by the exposure to mold and dampness (Table 6).

**Table 6:** Results of the multivariable regression models for the exposure to mold and damp as a risk factor for respiratory symptoms *Variable results are expressed as beta coefficients and 95% confidence intervals; otherwise, results are expressed as odds ratios and 95% CIs.

Parameter	OR	95% CI	p-value
Asthma	1.35	0.75, 2.38	0.312
Chest whistling or wheezing	1.74	1.13, 2.67	0.011
Chronic cough	1.32	0.87, 1.99	0.192
Productive cough	1.60	1.00, 2.57	0.051
Nasal symptoms without cold	1.37	0.91, 2.08	0.134
Use of asthma medications	1.06	0.46, 2.32	0.895
Cough over the past 12 months	0.99	0.65, 1.50	0.969
Dyspnea over the past 12 months	1.28	0.80, 2.04	0.303
Nocturnal chest tightness over the past 12 months	1.23	0.69, 2.16	0.481
Wheezing or whistling in the chest over the past 12 months	1.55	0.85, 2.77	0.144
Symptoms score*	0.19	-0.09, 0.47	0.088

## Discussion

The study investigated SHS exposure among citizens and residents living in KSA. In the participant sample, 67.3% (n=789) reported exposure to SHS; 40.3% (n=472) from home, 32.6% (n=382) from work colleagues, and 30.5% (n=357) from friends. Interestingly, a study in Qatar found that 19.3%(n=1219) reported smoking exposure at home, 3.1%(n=196) at the workplace or school, and 3.3%(n=204) in cafes and restaurants [11]. Another study in Malaysia found a prevalence of 57.8% (n=122), and restaurants were the most common place of SHS exposure, followed by workplaces and homes [12]. Also, a study in India found that 25.9% (n=67) were exposed to SHS inside the home and 37.5% (n=97) were exposed outside the home [13]. A study conducted in Thailand surveying 780 middle school students found that 46.8%(n=365) participants had SHS exposure at home [14]. A study among intermediate and secondary school students in Saudi Arabia found SHS of 32.7% (n=1049), 49.3% (n=1584), and 25% (n=801) outside, inside, and inside and outside the home, respectively [15]. This high frequency highlights the regularity of exposure within living environments. We can determine that most who suffer from SHS in Saudi Arabia are through members of the household, and the level of home exposure is high, like that of other countries. This makes sense as most smoking bans are usually in the workplace and public settings, increasing the likelihood of SHS exposure at home where no official bans exist.

Specifically focusing on SHS exposure at home, 76.9% (n=306) of the participants reported experiencing SHS almost daily. Daily exposure to SHS has been consistently linked to various adverse health effects, including respiratory issues such as asthma, impaired lung function, and an increased risk of lung cancer, cardiovascular diseases, and depression [16-18]. The CDC claims there is no ‘safe level’ of SHS exposure [4].

Furthermore, our study found a low prevalence of maternal regular smoking during the participants' childhood (n=13, 2.8%), while a relatively higher percentage indicated that their fathers were regular smokers during their childhood (n=201, 42.6%). A study in China found that about 75.1% of non-smoking pregnant women reported regular SHS exposure, the major source of which was their husbands [19]. In Thailand, a study found the main SHS sources were fathers [14].

Our study demonstrated that a large portion of participants (n=230, 48.7%) noted the presence of other regular smokers at home, including brothers (n=51, 26.7%), uncles (n=15, 7.9%), husbands (n= 2, 1.0%), and grandparents (n= 4, 2.1%). These results along with those from our study reflect the global issue of SHS home exposure especially through male members of the family, highlighting the importance of male education and empowerment regarding smoking cessation for the well-being of their families. By fostering a collective understanding of the hazards associated with SHS, families can work together to eliminate smoking within their homes, safeguarding the health of all household members, particularly children, from the harmful effects of SHS [20,21].

The present study also sought to evaluate whether respiratory symptoms were caused by external factors such as exposure to mold and dampness at home. Regarding exposure, the majority reported not having any kind of damage in their place of residence (n=985, 84.0%). Only 16.0%(n=187) indicated the presence of various types of damage, including dampness-related issues from walls, floors, or ceilings. While some studies failed to demonstrate a direct association between bio-aerosol concentrations and health effects in damp indoor environments [22], a meta-analysis conducted in 2011 found that dampness or mold was positively associated with multiple allergic and respiratory effects [23]. The CDC also states that mold exposure can cause respiratory symptoms such as wheezing and coughing in allergic individuals [24]. This is consistent with our study which found that exposure to mold and dampness was a significant risk factor for chest whistling or wheezing. Even without mold, dampness indoors can cause asthma attacks and other upper and lower respiratory problems, and exposure to mold has been linked to worsening of asthma, coughing, wheezing, nasal congestion, sore throat, sneezing, and rhinitis [25]. 

Furthermore, SHS exposure was a significant risk factor for an increased symptom score. Also, our study discovered that those exposed to SHS exhibited higher odds of reporting respiratory symptoms like asthma and wheezing. Our findings provide evidence that exposure to SHS is indeed a significant risk factor for the development of respiratory symptoms in adults for both short and long-term (up to 12 months). These results are consistent with a previous study on 20,421 adults from the Danish general population [26]. They found that second-hand smoke exposure in adulthood was associated with increased risks for dyspnea, wheezing, cough, and asthma, while exposure in childhood was associated with increased risks of wheezing and coughing in adults. Another study found that exposure to parental smoking in childhood increases the risk of persistent cough, chronic cough, and wheezing into young adulthood [27]. Likewise, a study in Norway on 8850 non-smokers found that daily SHS exposure was associated with productive cough and nocturnal dyspnea [10]. In the US, a study was conducted amongst truck drivers which also demonstrated a strong association between exposure to SHS and respiratory symptoms [28]. Altogether, these studies are consistent with our findings, which demonstrate that Saudi Arabia, like other countries, shows that SHS is a risk factor for developing respiratory symptoms.

Limitations

Our capacity to determine a cause-and-effect relationship between respiratory symptoms and SHS exposure was limited by using a cross-sectional study design. It was not possible to evaluate if the exposure occurred before the onset of symptoms using data gathered at a single time point. To provide more solid information about the association between respiratory symptoms and SHS exposure over time, longitudinal studies would be helpful. Additionally, the use of self-reported questionnaires to gauge exposure to secondhand smoke causes recall bias, which could affect self-reported statistics because individuals could not remember or specify their exposure levels precisely. Furthermore, the majority of participants in this study were females, which may alter the results as ideally the number of males and females should be equal. Additionally, the most common age group was young adults (18-30), which does not reflect SHS exposure and its effects on the middle-aged or elderly population. Finally, there were some missing records regarding certain parts of the questionnaire, which could affect the accuracy of the results.

Recommendations

We offer several recommendations for reducing SHS exposure, including targeted interventions at the household level promoting smoking cessation, and smoking cessation programs that should be made available to smokers through workplaces, healthcare providers, and other community organizations. Specifically, importance should be given to educating the public about the dangers of SHS at home, and should be tailored to different audiences, such as parents, young people, and healthcare professionals in Saudi Arabia. There should be emphasis on educating male household members specifically as they are the forerunners as sources of SHS according to most studies, including this one. These measures will serve to protect the health and well-being of the Saudi population and greatly reduce the impact of SHS on public health. Likewise, we recommend more studies in KSA take place exploring other effects of SHS on non-respiratory symptoms, including long-term effects such as cardiovascular disease, and in specific populations such as pregnant and children.

## Conclusions

To conclude, this study found that a significant percentage of the Saudi population is exposed to SHS. The majority of those exposed were through home exposure, most notably male family members. Furthermore, the study revealed a significant association between SHS exposure and the presence of respiratory symptoms. This study also found that mold and dampness exposure was a significant risk factor for wheezing, but not other respiratory symptoms.

The high prevalence of SHS exposure and its association with respiratory ailments demonstrate the urgent need for public awareness regarding its harms. Specifically, families and households should be targeted with an emphasis on male family members. Similarly, educational interventions are necessary amongst the general population as a means to assist them in avoiding SHS exposure. There is also a need for additional national research in this area on a larger scale in order to support the development of effective preventive policies to reduce SHS exposure. Eventually, these changes will serve to protect the general population in Saudi Arabia from the harmful effects of SHS, and awareness regarding its danger is the first step.
